# Variations in transcranial doppler among pediatric patients with sickle cell disease in the Brazilian Northeast: a cross-sectional study

**DOI:** 10.1016/j.htct.2026.106458

**Published:** 2026-04-19

**Authors:** Taciana Raulino de Oliveira Castro Marques, Suely Arruda Vidal, Heráclio Almeida da Costa, Ariani Impieri Souza

**Affiliations:** a Hospital Universidade Federal de Campina Grande (UFCG), Rua Carlos Chagas s/n, São José, Campina Grande, Paraíba, CEP: 58400-398, Brazil; b Instituto de Medicina Integral Prof. Fernando Figueira – IMIP, Rua dos Coelhos, 300, Boa Vista, Recife, Pernambuco, CEP: 50070-902, Brazil; c Imago – Diagnóstico por Imagem, Rua Dom Pedro II, 382, Centro, Campina Grande, Paraíba, CEP: 58400-565, Brazil

**Keywords:** Sickle cell disease, Transcranial doppler ultrasound, Stroke, Neurological manifestations

## Abstract

**Background:**

Stroke, the most severe neurological complication of sickle cell disease, can result in irreversible sequelae and death. Transcranial Doppler combined with periodic blood transfusions reduces the chances of stroke by 90 %. In Brazil, transcranial Doppler coverage for children is low (around 20 %). This study aimed to analyze the velocity of cerebral blood flow in pediatric patients using transcranial Doppler.

**Methods:**

This cross-sectional study analyzed 42 patients with sickle cell disease aged between 2 and 18 years who underwent transcranial Doppler following the Stroke Prevention Trial in Sickle Cell Anemia protocol. The time-averaged maximum mean velocities of the internal carotid and middle cerebral arteries were analyzed, considering biological, sociodemographic, and clinical factors.

**Results:**

The predominant genotype was hemoglobin SS, and the median age was 10 years (interquartile range: 7–13). Of the total, 42.9 % had never undergone the exam; 7.1 % had a history of stroke with a 66.7 % recurrence rate. Regarding transcranial Doppler, 83 % of patients had normal velocity, 2.4 % abnormal, 2.4 % conditional, 9.5 % low, and 2.4 % had inconclusive results. Higher time-averaged maximum mean velocities were observed in younger patients (*r* = -0.48; p-value = 0.001), in those with lower levels of hemoglobin (*r* = -0.37; p-value = 0.024), and with higher leukocyte counts (*r* = 0.33; p-value = 0.050). Stroke was associated with abnormal transcranial Doppler results (p-value < 0.001).

**Conclusion:**

Despite recommendations, primary stroke prevention is not yet effectively implemented in the northeast of Brazil, as evidenced by low transcranial Doppler screening rates and the high incidence of stroke in the study population.

## Introduction

Sickle cell disease (SCD) includes a group of autosomal recessive hereditary hemoglobinopathies characterized by a genetic mutation in the β-globin chain of hemoglobin, resulting in sickle hemoglobin (Hb S). The most common form is sickle cell anemia (Hb SS). The Hb SS and Hb Sβ⁰ genotypes are associated with the most severe clinical manifestations of the disease [1,2].

According to the National Neonatal Screening Program, the annual incidence of SCD in Brazil was 3.78 per 10,000 live births between 2014 and 2020, representing 1087 new cases. By 2022, the estimated prevalence ranged from 60,000 to 100,000 cases nationwide. This distribution is highly heterogeneous, with the highest incidences recorded in the states of Bahia, Piauí, and the Distrito Federal [3]. Additionally, in 2023, the state of Paraíba reported approximately 300 active cases [4].

The clinical manifestations of SCD are multisystemic, beginning in the early years of life and advancing to a broad range of acute and chronic complications that affect most organs and systems [1,2]. Neurological damage results from vasculopathy, hypercoagulability, thrombosis, hemolysis, and hypoxia [5,6]. Silent cerebral infarction, affecting 27 % of children over six years old, is the most common permanent neurological injury though it can be identified incidentally through nuclear magnetic resonance or screening [7].

Stroke, the worst neurological complication caused by SCD, is associated with motor and cognitive deficiencies, irreversible neurological sequelae, and death. The incidence of stroke in these individuals is 200-fold higher than in the general pediatric population [2,7,8]. Risk factors for ischemic and hemorrhagic stroke include low hemoglobin levels, leukocytosis, hypertension, previous strokes, acute chest syndrome, reticulocytosis, and increased lactate dehydrogenase (LDH). Blood transfusion, corticosteroid therapy, and nonsteroidal anti-inflammatory drugs are also potential risk factors for hemorrhagic stroke [8,9].

Following the Stroke Prevention Trial in Sickle Cell Anemia trial (STOP), a global screening protocol was established recommending annual transcranial Doppler (TCD) beginning at age 2 to monitor cerebral blood flow velocity (CBFV) and prevent stroke [10,11]. In Brazil, the Unified Health System (SUS) has offered this exam for patients with SCD aged from 2 to 16 years since 2012, prioritizing those with the Hb SS and Hb Sß genotypes [3,12]. TCD identifies patients with a higher risk of developing strokes, and actions, such as regular red blood cell transfusions, must be adopted thereby preventing it by 90 % of cases [10, 11, 12].

TCD coverage between 2018 and 2021 was 20.6 % in Brazil and 20.8 % in the northeastern rural regions [3,13]. Healthcare inequalities limit access to screening and diagnostic exams, leading to knowledge gaps on the patterns of illnesses in diverse populations, thereby complicating targeted hemotherapy [14,15].

Consequently, this study aimed to assess CBFV using TCD and identify its associated factors in socioeconomically vulnerable pediatric patients.

## Material and methods

This cross-sectional analytical study included pediatric patients aged 2 to 18 years diagnosed with SCD via isoelectric focusing or high-performance liquid chromatography. The inclusion criteria were the absence of red blood cell transfusions, fever, or acute clinical events within the four weeks preceding the TCD examination. The patients lived in Campina Grande, Paraíba, Brazil or neighboring municipalities and were under medical follow-up in the Alcides Carneiro University Hospital, a high-complexity public service integrated into the Federal University of Campina Grande. Data were collected from May 2022 to December 2024.

TCD was performed and analyzed by a single licensed radiologist. CBFV values of the internal carotid, middle cerebral, and basilar arteries were obtained. Moreover, biological (sex, age, and ethnic group), sociodemographic (residence, educational level, and family income), clinical (SCD genotype, regularity of outpatient follow-up, vaccination status, hospitalization in the last year, therapies, and history of stroke), and laboratory data (Hb levels, leukocyte count, reticulocyte count, and LDH) were collected via medical records and interviews using a specific questionnaire.

All patients underwent TCD following the Stroke Prevention Trial in Sickle Cell Anemia protocol [11]. A Samsung HS70 ultrasound device was used with a PA1–5A phased array transducer operating in a frequency range of 1.0–5.0 MHz. Assessments were conducted with patients awake, afebrile, and at least four weeks after clinical interventions including red blood cell transfusions.

The assessments focused on the time-averaged maximum velocities (TAMMV) of the internal carotid, middle cerebral, and basilar arteries. The transducer was positioned on the temporal and occipital acoustic windows to obtain images of the studied arteries. A color Doppler identified the points with the highest velocities, and the TAMMV of each artery was recorded at the point of interest. TCD results were classified as normal (TAMMV <170 cm/s), low conditional (TAMMV between 170 and 184 cm/s), high conditional (TAMMV between 185 and 199 cm/s), abnormal (TAMMV ≥200 cm/s), or inconclusive [10, 11, 12].

The SPSS software version 3.0 (IBM Corp., Armonk, USA) was used for statistical analysis. Data were presented as absolute and relative frequencies (categorical variables) or measures of central tendency and dispersion (numerical variables). The Chi-square test assessed the associations between categorical variables. Additionally, comparisons between an interval numerical variable and a categorical variable were assessed by Mood’s Median Test. Pearson’s correlation coefficient (r) was used to measure the intensity and direction of relationships between continuous numerical variables. Correlation values were classified as weak (0.10–0.29), moderate (0.30–0.49), or strong (0.50–1.0). A p-value of <0.05 was considered statistically significant for all tests.

The parents or caregivers of all patients signed an informed consent form, whereas patients over eight years old signed an informed assent form. This study was approved by the human research ethics committee of the Federal University of Campina Grande on August 10, 2022 (CAEE 60,830,422.4.0000.5182).

## Results

This study included all 42 patients with SCD aged between 2 and 18 years (median of 10 years: interquartile range: 7–13) receiving clinical follow-up at the referral center during the study period. The Hb SS genotype was found in 29 patients (69 %). Most patients were males (*n* = 27; 64.3 %), of African descent (*n* = 32; 92.9 %), and lived outside the reference area (*n* = 32; 76.2 %). Regarding guardians and caregivers (mostly mothers and fathers), 22 (52.4 %) reported nine years of formal education, and 31 (73.8 %) had a monthly family income of up to one minimum wage (Table 1).

**Table 1:** Biological, sociodemographic, and clinical characteristics of pediatric patients with sickle cell disease.

Variables	*n* = 42	%
**Genotype**		
Hb SS	29	69.0
Hb Sβ^+^	7	16.7
Hb Sβ^0^	4	9.5
Hb SC	2	4.8
**Age (years)**		
2 to 5	7	16.7
6 to 10	16	38.0
11 to 18	19	45.3
**Ethnic group**		
Of African descent	39	92.8
White	3	7.2
**Sex**		
Male	27	64.3
Female	15	35.7
**Educational level of caregivers**		
≤ 9 years	22	52.4
10 to12 years	15	35.7
>12 years	5	11.9
**Residency**		
Campina Grande	10	23.8
Other municipalities	32	76.2
**Family income (MW)**		
<1 minimum wage	9	21.4
1 minimum wage	22	52.4
>1 minimum wage	11	26.2
**Outpatient follow-up**		
Regular	20	47.6
Irregular	22	52.4
**Vaccination schedule**		
Complete	16	38.0
Incomplete	26	62.0
**Ischemic Stroke**	3	7.1

*Minimum wage = *R*$ 1302.00 (2022 – approximately $253.00 USD).

Over half the patients had inconsistent medical follow-up (*n* = 22; 52.4 %) and presented incomplete vaccination records (*n* = 26; 62 %). Also, 33 (78.5 %) were taking hydroxyurea regularly for more than one year with doses ranging from 20–35 mg/kg/day. Eight patients were not receiving hydroxyurea due to therapeutic decisions (seven had never been prescribed the drug, one had the treatment discontinued by the hematologist), and one patient independently discontinued the treatment. A total of 26 patients had previously received red blood cell transfusions, and two reported chronic transfusion therapy indicated for secondary stroke prevention; these patients received their last red blood cell transfusion more than four weeks prior to the TCD conducted in this study.

Three patients (7.1 %), all with the Hb SS genotype, had experienced a total of five ischemic strokes. Two of these patients had stroke recurrence despite being enrolled in a secondary prevention program. The third patient was not on a transfusion protocol, and the stroke was identified during the study interview, when significant right-sided hemiparesis was noted. From that point on, appropriate clinical and therapeutic measures were taken. The age of the patients at the time of the stroke ranged from 7–11 years old (median age of 7 years and 9 months).

In 2024, 30 patients (71.4 %) were hospitalized, representing a total of 63 admissions. The most common causes of hospitalization were pain crises, infections, and acute chest syndrome. Of the neurological complaints, 25 patients (59.5 %) reported chronic headaches, and two (4.7 %) had epilepsy. No deaths occurred during the study period.

TCD results showed increased TAMMV in the middle cerebral and internal carotid arteries in 66.7 % and 26.2 % of the patients, respectively. The TAMMV ranged from 61.6–221.2 cm/s, with a mean of 119.5 ± 30.8 cm/s (raw data).

Before this study, 18 (42.9 %) patients aged 2–17 years, 13 (72.2 %) of whom were over six years of age, had never undergone a TCD, and only three (7.1 %) performed the exam regularly. Thirty-five (83 %) patients had normal results; most of those with abnormal, conditional or low velocity results had the Hb SS genotype (*n* = 5/6; 83.3 %). Although no statistical significance was observed (p-value = 0.055), higher TAMMV were observed in genotypes associated with the most severe forms of the disease (Hb SS and Hb Sβ^0^) (Table 2).

**Table 2:** Transcranial Doppler and TAMMV of cerebral blood flow in pediatric patients with sickle cell disease according to genotype.

Genotype	Hb SS	Hb Sβ^+^	Hb Sβ^0^	Hb SC	TOTAL
TCD	n ( %)	n ( %)	n ( %)	n ( %)	n ( %)
Normal	23 (79.3)	7 (100.0)	4 (100.0)	1 (50,0)	35 (83.3)
Abnormal	1 (3.4)	0	0	0	1 (2.4)
Conditional	1 (3.4)	0	0	0	1 (2.4)
Low velocity	3 (10.3)	0	0	1 (50.0)	4 (9.5)
Inconclusive	1 (3.4)	0	0	0	1 (2.4)
**Total**	**29 (100)**	**7 (100)**	**4 (100)**	**2 (100)**	**42 (100)**
**TAMMV**					p-value*
**Median (cm/s)**	119.5	93.7	137.9	110.0	0.055

⁎ Mood's Median Test. Statistically significant differences among groups if p-value <0.05. TCD: Transcranial Doppler; TAMMV: time-averaged median maximum velocity of cerebral blood flow.

**Figure 1:**
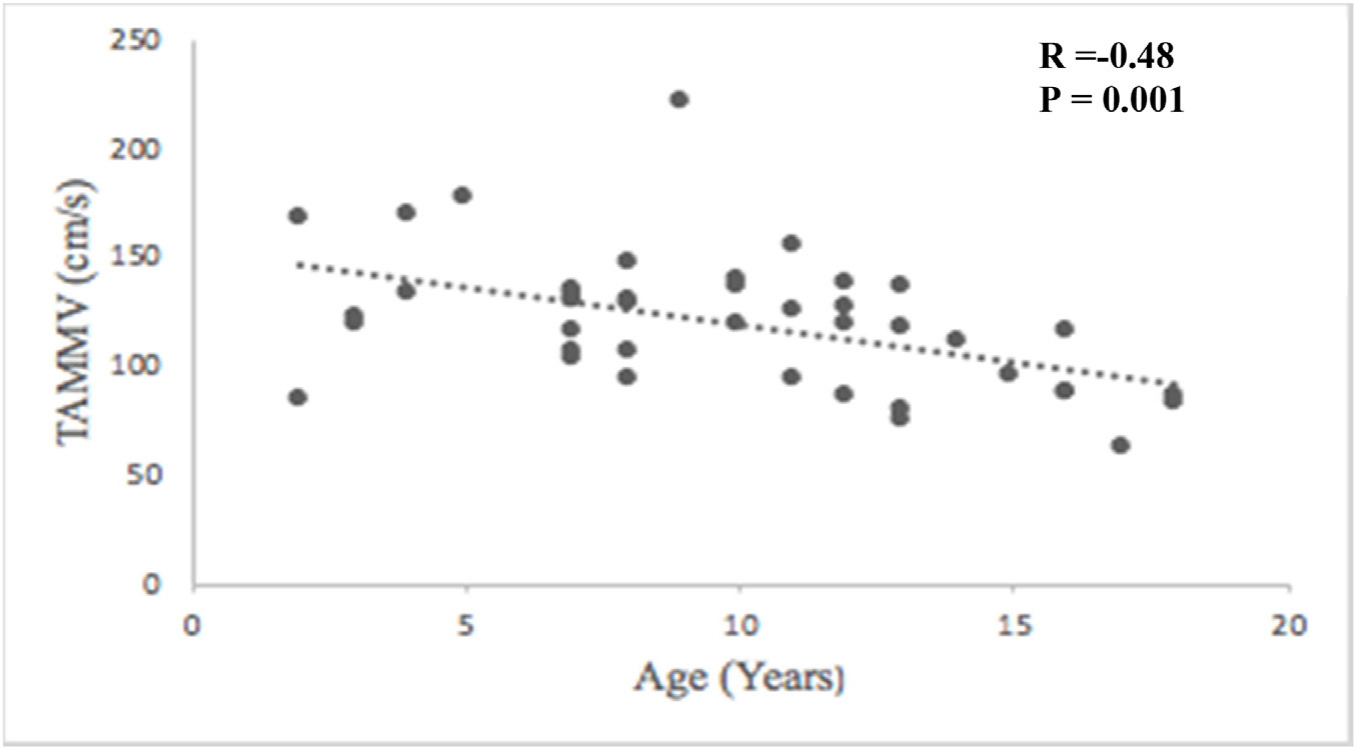
Pearson correlation between age and TAMMV of cerebral blood flow in pediatric patients with sickle cell disease.

Age progression was associated with a reduction of TAMMV (*r* = −0.48; p-value = 0.001) (Figure 1). No association was found between TAMMV and therapeutic interventions, hospitalizations, or outpatient follow-up. Previous stroke was associated with abnormal TCD results (p-value <0.001) (Table 3).

TAMMV: time-averaged median maximum velocity

**Table 3:** Clinical characteristics of pediatric patients with sickle cell disease and associations with transcranial Doppler results and TAMMV of cerebral blood flow.

Variable	TAMMV	p-value	TCD		p-value
	Median		Normal N ( %)	Other* N ( %)	
**Use of hydroxyurea**					
Yes	115.8	0.133	26 (78.8)	7 (21.2)	0.130
No	130.2		9 (100)	0	
**Hospitalization in the previous year**					
Yes	121.1	0.733	23 (76.6)	7 (23.4)	0.067
No	109.0		12 (100)	0	
**Headache**					
Yes	109.2	0.122	18 (90)	2 (10)	0.269
No	129.7		17 (77.2)	5 (22.8)	
**ASA**					
Yes	110.9	0.694	5 (62.5)	3 (37.5)	0.079
No	121.1		30 (88.2)	4 (11.8)	
**Regular blood transfusions**					
Yes	117.0	0.751	22 (84.6)	4 (15.4)	0.776
No	127.5		13 (81.2)	3 (18.8)	
**Vaccination record**					
Updated	126.8	0.340	14 (87.5)	2 (12.5)	0.570
Outdated	117.0		21 (80.7)	5 (19.3)	
**Follow-up**					
Regular	126.1	0.354	17 (85)	3 (15)	0.782
Irregular	116.6		18 (81.8)	4 (18.2)	
**Stroke**					
Yes	155.0	0.999	0	3 (100)	< 0.001*
No	118.8		35 (89.7)	4 (10.3)	

Mood's Median Test: Statistically significant differences between groups if p-value < 0.05.

TAMMV: time-averaged maximum mean velocity; TCD: Transcranial Doppler (Abnormal, conditional, inconclusive); ASA: Acetylsalicylic acid.

Regarding laboratory variables, higher TAMMV values were associated with lower Hb levels (*r* = −0.37; p-value = 0.024) and higher leukocyte counts (*r* = 0.33; p-value = 0.050) (Table 4). Further, patients with Hb SS showed the lowest median Hb levels (p-value = 0.038) and highest LDH levels (p-value = 0.032) when compared with the other patients (Table 5).

**Table 4:** Pearson correlation between TAMMV of cerebral blood flow and laboratory variables in pediatric patients with sickle cell disease.

	TAMMV	p-value
**Hemoglobin**	**−0.37**	**0.024**
**Leukocytes**	**0.33**	**0.050**
**LDH**	0.16	0.415
**Reticulocytes**	−0.01	0.972
**Platelets**	−0.04	0.815

*LDH: lactate dehydrogenase; TAMMV: time-averaged maximum mean velocity (cm/s).

**Table 5:** Associations between medians of laboratory variables and genotypes of pediatric patients with sickle cell disease.

	Hb SS *n* = 29	Hb Sβ^0^ *n* = 4	Hb Sβ^+^ *n* = 7	Hb SC *n* = 2	p-value
**Hemoglobin (g/dL)**	7.55	9.15	9.80	8.60	**0.038**
**Leukocytes (x 10^9^/L)**	13.60	10.65	8.20	8.56	0.127
**LDH (U/L)**	1187.0	639.5	412.5	483.0	**0.032**
**Reticulocytes ( %)**	7.26	6.61	6.75	3.09	0.149
**Platelets (x 10^9^/L)**	390	486	331	276	0.909

Mood's Median Test: Statistically significant differences between groups if p-value < 0.05.

LDH: lactate dehydrogenase.

## Discussion

This study analyzed the TCD results of 42 pediatric patients (aged 2–18 years) with SCD. The cohort was predominantly male, of African descent, and of low socioeconomic status, with the Hb SS genotype being the most prevalent. The frequency of stroke (7.1 %) was consistent with literature for patients without appropriate screening, which is 5–17 % during childhood and adolescence [8,13].

Stroke in patients with SCD is associated with inadequate implementation of primary care programs, therapeutic interventions, and inconsistencies in regular TCD screenings. Although most patients in this study were older than six years, nearly half had never undergone a TCD. This finding highlights the limited access to primary prevention during early childhood [14,16].

A Brazilian study showed that almost one-third of children had never performed a TCD, and among them, the prevalence of stroke was 4.4 % [15]. Late and irregular screening for stroke was also observed in a recent national study [17]. In studies conducted in Spain and France, the median age for the first TCD ranged between two and three years, with over 80 % occurring before three years of age [18,19]. These findings indicate the great discrepancy in access to TCD worldwide [3,18, 19, 20].

Stroke screening patterns and risk interventions differ between regions. In scenarios where guidelines are not properly implemented, it is unclear how specialists assess patients and apply TCD results. In these scenarios, deviations from guidelines and missed opportunities for stroke prevention have been observed [14,17,21].

Factors hampering access to primary prevention include patient-related barriers (geographic location, poor adherence, low socioeconomic status, caregiver unawareness, and fear of blood transfusions) and health system limitations (inconsistent TCD availability, a shortage of specialists and equipment, and provider unfamiliarity with prevention guidelines) [15,17,20, 21, 22].

In this study, most caregivers attributed the lack of screening to the distance between their residence and the referral center, scheduling difficulties, and a lack of transportation or awareness of primary stroke prevention. Although regular outpatient follow-up and prior TCD examinations correlate with better adherence, the annual rate of missed opportunities for TCD screening remains between 61 % and 88 %. Furthermore, increasing age is associated with a lower likelihood of undergoing the exam [20].

Great variations in occurrence rates among different TCD categories were observed. In this study, the frequency of abnormalities was 2.4 %; however, studies have shown higher (4.8 % to 17 %) [15,18,23,24], lower (1.2 % to 2.1 %), and similar frequencies to our study [17,25,26]. Abnormal TCD results have been documented, including in the Brazilian Northeast, suggesting a lower risk of stroke and the possibility of effective treatments and interventions to prevent cardiovascular diseases [27,28].

Studies have shown divergences in lower TCD flow rates (ranging from 1 % to 8.4 %) [15,18,23,26,28], while a rate of 9.5 % was observed in the current study. This result may be associated with cardiovascular diseases and reflect the complete occlusion of a previous stenotic artery with the development of collateral circulation, thereby, decreasing blood flow in the main artery [28]. Considering conditional TCD, the literature shows a higher prevalence than in this study [15,18,23, 24, 25, 26,28].

Several factors may have contributed to these differences: heterogeneity of the genotypes, use of hydroxyurea or other therapeutic interventions, access to screening and diagnostic tools, methodological limitations of different studies, and experience of the operators performing and interpreting the exam [15,20,28]. Environmental variables, such as climate, seasonality, and nutritional deficiencies, may have affected the results too [29].

Associations with previous stroke and abnormal TCD categories were observed. A study involving children with SCD in Jamaica observed an association between abnormal TCD results and previous stroke [29]. This variation is related to progressive vasculopathy, with severe arterial stenosis or Moyamoya syndrome, contributing to further cerebrovascular events. Although patients were enrolled in secondary prevention through chronic transfusion therapy, the recurrence rate observed in this study (66.7 %) was comparable to rates reported in untreated populations (60 % to 92 %) [22].

The TAMMV showed a significant negative correlation with age. Although not statistically significant, higher TAMMV were often observed in patients with Hb SS and Hb Sß^0^. These findings corroborate literature showing that increased CBFV is associated with a high risk of stroke, mainly in the most severe forms of the disease and during the first decade of life [8,17,25]. Correlations between TAMMV, genotypes, and age are well-known in pediatric patients with SCD [15,26, 27, 28, 29].

The hemodynamics of cerebral blood flow account for the higher TAMMV values observed in younger patients. Newborns show a TAMMV of 24 cm/s in the middle cerebral arteries; this velocity increases gradually until four to six years of age, when it reaches its peak and then a gradual decrease is observed after 10 years of age [30].

Higher TAMMV were associated with lower Hb levels and higher leukocyte counts, which showed a weak positive correlation with LDH levels and no correlation with the reticulocyte count. As expected, the lowest Hb levels and highest LDH levels were observed in patients with Hb SS [29].

Anemia and the effects of severe hemolysis (reduced vascular smooth muscle relaxation, activation of adhesion molecules, increased platelet adhesion and aggregation, increased inflammatory processes, progressive vasculopathy, and compensatory mechanisms for cerebral perfusion) might explain the correlation between reduced Hb levels and increased TAMMV [6,17,28].

The positive correlation between TAMMV and leukocyte counts is linked to chronic inflammation, as leukocytes adhere to other cells and the vascular endothelium, promoting intimal hyperplasia, smooth muscle proliferation, vasculopathy, and subsequent ischemia [6].

Other studies have highlighted the role of reticulocytosis in elevating TAMMV. Reticulocytes released into the circulation express surface adhesion molecules that trigger a cascade leading to cerebral vasculopathy, thereby contributing to impaired cerebral rheology and vascular occlusion [6,29]. Additionally, none of the hemolysis markers are highly specific and may vary depending on clinical conditions (iron deficiency, inflammation, and variations in bone marrow) [5].

The results of this study should be cautiously interpreted since this research was conducted in a single center even though this center is a referral hospital for children with SCD.

## Conclusion

This study demonstrated that higher TAMMV were associated with younger age, lower hemoglobin levels, and higher leukocyte counts, while abnormal TCD velocities were linked to a history of stroke. These findings underscore the critical role of TCD in stroke risk stratification for pediatric patients with SCD. Despite current guidelines, primary stroke prevention remains suboptimally implemented in the Brazil Northeast, characterized by high rates of missed evaluations and stroke incidence. Factors such as regularity of follow-up, referral efficiency, and socioeconomic status significantly impact adherence. Targeted interventions are essential to address these barriers and expand TCD access, thereby improving stroke prevention in this vulnerable population.

## Data availability

The data that support the findings of this study are available from the corresponding author upon reasonable request.

## Authors’ contributions

Taciana Raulino de Oliveira Castro Marques: study conception and design, data acquisition, data analysis, and interpretation, paper composition. Suely Arruda Vidal: study conception and design, results revision**,** paper revision, approval of the final version. Heráclio Almeida da Costa: exams conduction, results description and analysis, critical review, and approval of the final version. Ariani Impieri Souza: study conception and design, results revision**,** paper revision, approval of the final version.

## Conflicts of interest

none.
